# Applying the information–motivation–behavioral model to explore the influencing factors of self-management behavior among osteoporosis patients

**DOI:** 10.1186/s12889-020-8292-x

**Published:** 2020-02-06

**Authors:** Lhakpa Tsamlag, Huwen Wang, Qiuming Shen, Yue Shi, Shuxian Zhang, Ruijie Chang, Xiyu Liu, Tian Shen, Yong Cai

**Affiliations:** Department of Community Health and Behavioral Medicine, School of Public Health, Shanghai Jiao Tong University School of Medicine, 227 South Chongqing Road, Shanghai, 200025 China

**Keywords:** Osteoporosis, self-management behaviors, information, Motivation, Behavioral skills model (IMB)

## Abstract

**Background:**

The prevalence of osteoporosis (OP) is rapidly increasing. Healthy behaviors are crucial for the management of OP. Application of the information–motivation–behavioral skills (IMB) model has been verified in various chronic diseases, but this model has not been investigated for behavioral interventions among people with OP. This study aimed to examine factors influencing OP self-management behavior and their interaction paths based on the IMB model.

**Methods:**

We conducted a cross-sectional study using a convenience sampling method in 20 community health service centers in Shanghai, China. Predictive relationships between IMB model variables and self-management behaviors were evaluated using an anonymous questionnaire. Structural equation modeling was used to test the IMB model.

**Results:**

In total, 571 participants completed the questionnaire, of which 461 (80.7%) were female. Participants’ mean age was 68.8 ± 10.1 years. Only 101 (17.7%) participants were classified as having better OP self-management behaviors. The model demonstrated the data had an acceptable fit. Paths from information to self-efficacy (β = 0.156, *P* < 0.001) and self-management behaviors (β = 0.236, *P* < 0.001), from health beliefs to self-efficacy (β = 0.266, *P* < 0.001), from medical system support to self-efficacy (β = 0.326, *P* < 0.001) and self-management behaviors (β = 0.230, *P* < 0.001), and from self-efficacy to self-management behaviors (β = 0.376, *P* < 0.001) were all significant and in the predicted direction.

**Conclusion:**

This study validated the utility of the IMB model for OP self-management behaviors in this population. Middle-aged and older adult patients with OP have poor self-management behaviors. Enhanced knowledge about OP and is important for improving self-management behaviors.

## Background

Osteoporosis (OP) is a systemic skeletal disease characterized by low bone mass and microarchitectural deterioration of bone tissue, with consequent increases in bone fragility and susceptibility to fracture [1]. With the gradual acceleration of population aging, the prevalence of OP in China is increasing each year. A meta-analysis showed that the prevalence of OP in China was 14.94% in 2008, but rose to 27.96% in 2015 [2]. Chronic pain caused by OP seriously interferes with normal daily activities. Mortality exceeds 20% within 6–12 months after a fracture [3], and the disease is associated with a huge economic burden that is expected to increase significantly over coming decades [4]. Given the long-term health damage and large economic losses associated with OP, there is an urgent need to improve the prognosis of OP. No ideal pharmacological agent has been identified for the treatment of OP [5], but safe and effective medications are available to reduce the risk for fractures [6]. However, OP medication adherence remains a major problem. One review that included 124 studies [7] reported the prevalence of medication adherence ranged from 12.9 to 95.4%. Ross et al. [8] reported that fracture risk increased by approximately 30% with medication noncompliance and by 30–40% with non-persistence. Low medication adherence was also associated with a 37% increase in the risk for all-cause hospitalization [9]. The relative risk reduction at 12 months for hip fracture was 60% for persistent compared with non-persistent patients [10].

OP is a chronic disease, like diabetes and hypertension [11]. Management of most chronic illnesses is characterized by extensive responsibility assumed by patients [12]. Barlow [13] defined self-management as an individual’s ability to manage the symptoms, treatment, physical and psychosocial consequences, and lifestyle changes inherent in living with a chronic condition. Improvement of self-management behaviors (e.g., exercise and diet) and cognitive behaviors (e.g., effective coping) are primary focus areas for these types of disease interventions [14]. Participation in self-management behaviors is seen as the proximal outcome that influences the long-term distal outcome of improved health status [15].

Modification of health behaviors requires consideration of factors that influence healthy behaviors [16]. A previous qualitative study showed that pre-requisites for OP patients to adopt self-care behaviors included increasing their hope of living longer, physician’s attention to the patient’s needs, media promotion, and family support [17]. Other studies have found close relationships between OP knowledge, health beliefs, self-efficacy, and health behaviors [18–20]. As a predictor, self-efficacy is supposed to facilitate the formation of behavioral intentions, development of action plans, and initiation of actions [21]. Although knowledge is often considered insufficient for behavioral change, information about OP may be important in developing OP self-management behaviors [22]. Health belief constructs vary in their effectiveness as predictors of behavior [23]. A meta-analysis of health belief studies found that susceptibility and perceived barriers were the most powerful behavioral influences [24]. Social support has also been noted as critical to the success of a person’s ability to self-manage [12].

Application of theory-based health behavior change models is the current trend in OP self-management education. This is because systematic models are more likely to effectively change behaviors and maintain behavioral changes than health information alone [25]. There are several models for the prevention of fragility fractures and treatment of OP, including the health belief model [26], knowledge attitudes and practices model [27], social cognitive theory [28], and motivational interviewing [29]. A theoretical model that has received widespread attention in the literature is the information–motivation–behavioral skills (IMB) model, which is based on a critical review and integration of constructs from several theories of health behavior. The IMB model was first applied with high-risk groups (e.g., people with AIDS) and has gradually been applied to those with chronic diseases such as diabetes [30]. The IMB model is useful in explaining factors that influence healthy behavior.

Fisher and Fisher [31] first proposed the IMB model in 1992. Compared with previous health-related behavior change models, the IMB model draws on the understanding of “motivation” from rational behavior theory, and introduces the concept of “self-efficacy” drawn from social cognitive theory. Potential factors are summarized in three components: information, motivation, and behavioral skills [32]. Information refers to accurate behavior-specific knowledge. Motivation is defined as an integrated function of personal motivation and social motivation. Personal motivation reflects an individual’s attitude or belief, and social motivation rests on their perception of social support. Behavioral skills include an individual’s self-efficacy and objective skills for performing a behavior [33]. The IMB model of adherence [34] suggests that adherence information and motivation often covary, but adherence motivation may be present when adherence information is inaccurate or insufficient, and vice versa [21]. If individuals have sufficient behavior-specific information, they are more inclined to build behavior skills and motivation and then to engage in the targeted health behavior [33]. However, information and motivation may also directly affect behavior when complex behavioral skills are not required for the performance of the behavior [35].

To our knowledge, no studies have investigated the IMB model for an OP behavioral intervention. This study aimed to explore factors influencing OP self-management behaviors and their interaction paths based on the IMB model. Understanding the relationships among the cognitive, emotional, and behavioral skills factors that influence healthy behaviors for OP in the IMB model may support the development and implementation of evidence-based interventions for OP in the community.

## Method

### Study site

We conducted this cross-sectional study in December 2016. We recruited patients from 20 community health service centers in Shanghai, China, using a convenience sampling method. These 20 primary healthcare centers included 10 urban and 10 suburban areas, and each center had an OP clinic. Community health service centers provide free bone mineral density (BMD) testing and professional guidance from specialist OP doctors.

### Inclusion criteria

The inclusion criteria were: 1) peak BMD value measured by a dual-energy X-ray absorption (DXA) detector lower than the standard reference population by at least 2.5 standard deviations or more (T-score ≤ 2.5); 2) provided informed consent and willing to cooperate with the research; and 3) aged ≥45 years.

### Recruitment and procedure

With the help of the Community Health Center, our investigators carried out face-to-face interviews with the patients who volunteered to participate in this study. Before filling in the questionnaire, the investigator fully explained the purpose of the survey to the participants and emphasized the protection of privacy. The questionnaire covered information about participant demographics and the constructs of the IMB model. The questionnaire took 30 min to complete. Participants were compensated 50 RMB (approximately 8 USD) for their participation after completion. This study was reviewed and approved by the Ethics Committee of the School of Public Health, Shanghai Jiao Tong University.

### Sampling size

In the structural equation modeling studies, the sample size should be at least 100, preferably 200 or more [36]. If the number of samples is analyzed from the number of observed variables in the model, the ratio between the number of samples and the number of observed variables is at least 10:1 to 15:1 [37]. Based on the number of observed variables and the actual situation of the community, we recruited 600 patients, with 30 patients included from each center. 600 participants were recruited to the study, of whom 571(95.2%) completed the questionnaire adequately for further analysis.

## Measures

### Basic data

The collected demographic characteristics included participants’ self-reported sex, age, body mass index, highest education level, current marital status, income, family history of OP, OP duration, calcium supplement use, and recent BMD measurements. (Please see Additional file 1 for the questionnaire.)

### Information: OP knowledge

Information on OP knowledge was measured using the 32-item version of the OP Knowledge Test (OKT). This test has two subscales: nutrition (items 1–11 and 18–32) and exercise (items 1–17 and 30–32). Response options for each item were “Right,” “Wrong,” or “I don’t know.” Items were scored as correct or incorrect, with correct answers coded as 1 and incorrect answers coded as 0. A higher score indicates a higher level of information. The OKT demonstrated internal consistency (Cronbach’s alpha coefficients: total scale = 0.859, nutrition subscale = 0.839, and exercise subscale = 0.781).

### Motivation: personal motivation and social support

#### Personal motivation: health beliefs

This part of the questionnaire used the 42-item version of the OP Health Belief Scale (OHBS) [38], which includes seven subscales: susceptibility (items 1–6); seriousness (items 7–12); benefits exercise (items 13–18); benefits calcium intake (items 19–24); barriers exercise (items 25–30); barriers calcium intake (items 31–36); and health motivation (items 37–42). The OHBS is scored by awarding points based on responses to each item (e.g., “Strongly Agree” = 5 points to “Strongly Disagree” = 1 point). Because there were six items in each subscale, the possible score for each subscale was 6–30, and the possible total score was 42–210. Test-retest reliability for the total instrument was 0.90, with the subscale reliabilities ranging from 0.778 to 0.920.

#### Social support: medical system support

We measured community medical system support for patient self-management behaviors. Patient compliance and understanding has been linked to the quality of physician explanations and the physician-patient relationship [39]. This part of the questionnaire included 11 items, with responses on a 5-point Likert scale (“Strongly Disagree” to “Strongly Agree”). The Cronbach’s alpha coefficient was 0.960.

#### Behavioral skills: self-efficacy

This part of the questionnaire used the 21-item version of the OP Self-Efficacy Scale (OSES) [40], which was developed as a measure of self-efficacy (or confidence) in behaviors related to physical activity and calcium intake [41]. The instrument comprises an exercise subscale (items 1–10) and a calcium intake subscale (items 11–21). The scoring method for the scale is an 11-point scoring method from 0 (“No Confidence At All”) to 10 (“Full Confidence”). A higher score represents stronger self-efficacy. In the present study, the reliability coefficient for the internal consistency of the total tool was 0.97, and those for the exercise and calcium intake subscales were 0.973 and 0.968, respectively.

#### Behavior: self-management behaviors

This section of the questionnaire used the Patient OP Self-Management Scale, which covers nutrition, exercise, and treatment-related behaviors. The Chinese version of this tool was validated by Shen et al. [42]. The scale uses a 5-point Likert scoring method. The patient selects the response option corresponding to the frequency of completing various behaviors in the past 30 days. A larger score indicates higher frequency, with a score of 1 point meaning “Never” and a score of 5 points meaning “Frequent.” The overall Cronbach’s alpha coefficient for this scale was 0.889, and those for the dimensions were 0.812–0.928. In this study, a score of 4 points or above was defined as “better self-management behaviors.”

### Statistical analyses

Data were collected using Epidata 3.1 software (EpiData Association, Odense, Denmark). We use the mean or median of the result in the rest of the samples to replace the missing values (missing data< 1%).After data entry and checking, data analyses were performed with SPSS version 20.0 (IBM Corp. Released 2013. IBM SPSS Statistics for Windows, Version 22.0. Armonk, NY: IBM Corp). Continuous variables were described as mean ± standard deviation (SD), and binary and categorical variables were described as frequency (percentage). The level of statistical significance was set at 0.05 for all analyses. We used linear regression to analyze the relationships between demographic characteristics and self-management behaviors (univariate analysis). In the examination of relationships between influencing factors and self-management behaviors, each impact factor was analyzed using linear regression. When investigating the paths of self-management behaviors, AMOS 23.0 (Arbuckle, J. L. (2014). Amos (Version 23.0). Chicago: IBM SPSS) was used to analyze the paths in the recursive model. Mediation effects were tested using a bias-corrected bootstrap method in AMOS 23.0.

## Results

### Sociodemographic characteristics

Table 1 presents participants’ basic sociodemographic information. Of the 571 participants, 110 (19.3%) were male and 461 (80.7%) were female. Participants’ mean age was 68.8 ± 10.1 years, 133 (23.3%) participants had a primary school or below education, and 382 (66.9%) had a monthly income > 3000 RMB. In addition, 110 (18.4%) participants had family history of OP, 469 (78.6%) had been diagnosed with OP in the past 5 years, 377 (63.1%) had present use of calcium supplements, and 140 (23.5%) had no BMD measurements within 1 year.

**Table 1:** Participants’ sociodemographic characteristics and their associations with osteoporosis self-management behaviors (*N* = 571)

Socio-demographics	Number of participants N (row%)	β (95%CI)
Gender		
Male	110(19.3%)	1
Female	461(80.7%)	2.28 (0.394–4.166)*
Age group (years)		
45–59	60(10.5%)	1
60–74	348(60.9%)	0.110 (−0.422–4.520)
≥ 75	163(28.5%)	−0.046 (−3.603–1.735)
BMI (kg/m2) ^a^		
< 18.5	17(03.0%)	−0.585 (−5.032–3.386)
18.5–23.9	328(57.4%)	1
24–27.9	192(33.6%)	−0.507 (−2.132–1.117)
≥ 28	34(06.0%)	−1.291 (−4.512–1.930)
Highest education level		
Primary or below	133(23.3%)	1
Middle school	192(33.6%)	5.166 (3.236–7.095)***
Senior high school	148(25.9%)	6.253 (4.209–8.297)***
College degree or above	98(17.2%)	7.395 (5.117–9.672)***
Current marital status		
Married	504(88.3%)	1.184 (−1.136–3.505)
Single, divorced or widowed	67(11.7%)	1
Income		
< 3000	189(33.1%)	1
3000–6000	344(60.2%)	2.544 (0.947–4.141)**
> 6000	38(06.7%)	2.571 (1.003–4.140)**
Had family history of OP		
No	466(81.6%)	1
Yes	105(18.4%)	2.802 (0.887–4.718)***
OP duration (years)		
< 5	445(77.9%)	1
5–9	87(15.2%)	0.739 (−1.345–2.823)
≥ 10	39(06.8%)	1.869 (0.385–3.354)*

*P < 0.05, **P < 0.01, ***P < 0.001

^a^body mass index

The linear regression analyses showed that five sociodemographic characteristics had a statistically significant impact on OP self-management behavior. These were: female sex (β = 2.28, 95% confidence interval [6]=0.394–4.166); high education level (β = 7.395, 95% CI = 5.117–9.672); high income (β = 2.571, 95% CI = 1.003–4.140); family history of OP (β = 2.802, 95% CI = 0.887–4.718); and OP diagnosis for ≥10 years (β = 1.869, 95% CI = 0.385–3.354).

### Summary statistics of model variables

The descriptive information for each measure is presented in Table 2. The mean ± SD values were 15.85 ± 6.18 for the OKT total score, 147.33 ± 17.91 for the OHBS total score, 43.53 ± 7.85 for the medical system support score, and 127.55 ± 40.53 for the OSES total score. The linear regression analysis showed that these four variables had statistically significant effects on OP self-management behavior.

**Table 2:** Model variables associated with osteoporosis self-management behaviors (*N* = 571)

Variables		Range	X¯ ± *SD*	Adjustedβ (95%CI)
Information	OP Knowledge Test (OKT)	0~32	15.85 ± 6.18	0.65 (0.54–0,75) ***
	Nutrition subscale	0~26	8.62 ± 4.08	0.82 (0.69–0.95) ***
	Exercise subscale	0~20	13.26 ± 5.11	0.83 (0.66–1.00) ***
Motivation--Personal	OP Health Belief Scale (OHBS)	42~210	147.33 ± 17.91	0.22 (0.18–0.25) ***
	Susceptibility	6~30	20.65 ± 4.25	0.38 (0.20–0.55) ***
	Seriousness	6~30	19.30 ± 4.67	0.21 (0.04–0.36) **
	Benefits Exercise	6~30	22.70 ± 4.13	0.76 (0.59–0.93) ***
	Benefits Calcium Intake	6~30	22.09 ± 4.33	0.47 (0.30–0.64) ***
	Barriers Exercise	6~30	20.26 ± 3.85	0.63 (0.44–0.81) ***
	Barriers Calcium Intake	6~30	20.26 ± 3.92	0.63 (0.45–0.81) ***
	Health Motivation	6~30	22.07 ± 4.27	0.93 (0.77–1.09) ***
Motivation--Social	Medical system support	11~55	43.53 ± 7.85	0.56 (0.48–0.65) ***
Behavioral skills	OP Self-Efficacy Scale (OSES)	0~210	127.55 ± 40.53	0.13 (0.11–0.14) ***
	Exercise subscale	0~100	54.64 ± 22.14	0.21 (0.18–0.24) ***
	Calcium Intake subscale	0~110	72.91 ± 24.09	0.19 (0.16–0.22) ***
Behavior	OP Self-management behaviors	11~55	34.73 ± 9.09	

95% CI: 95% confidence interval; Adjusted β: the results of the linear regression analysis were standardized and adjusted for gender, education, income, family history, OP duration

*P < 0.05, **P < 0.01, ***P < 0.001

The mean ± SD for the OP self-management behaviors score was 34.73 ± 9.09, and 101 (17.7%) participants were classified as having better OP self-management behaviors. Among these participants, 87 (15.2%) participants had better self-management behaviors in the nutrition dimension and 122 (21.4%) in the exercise dimension.

### Model testing

**Fig. 1:**
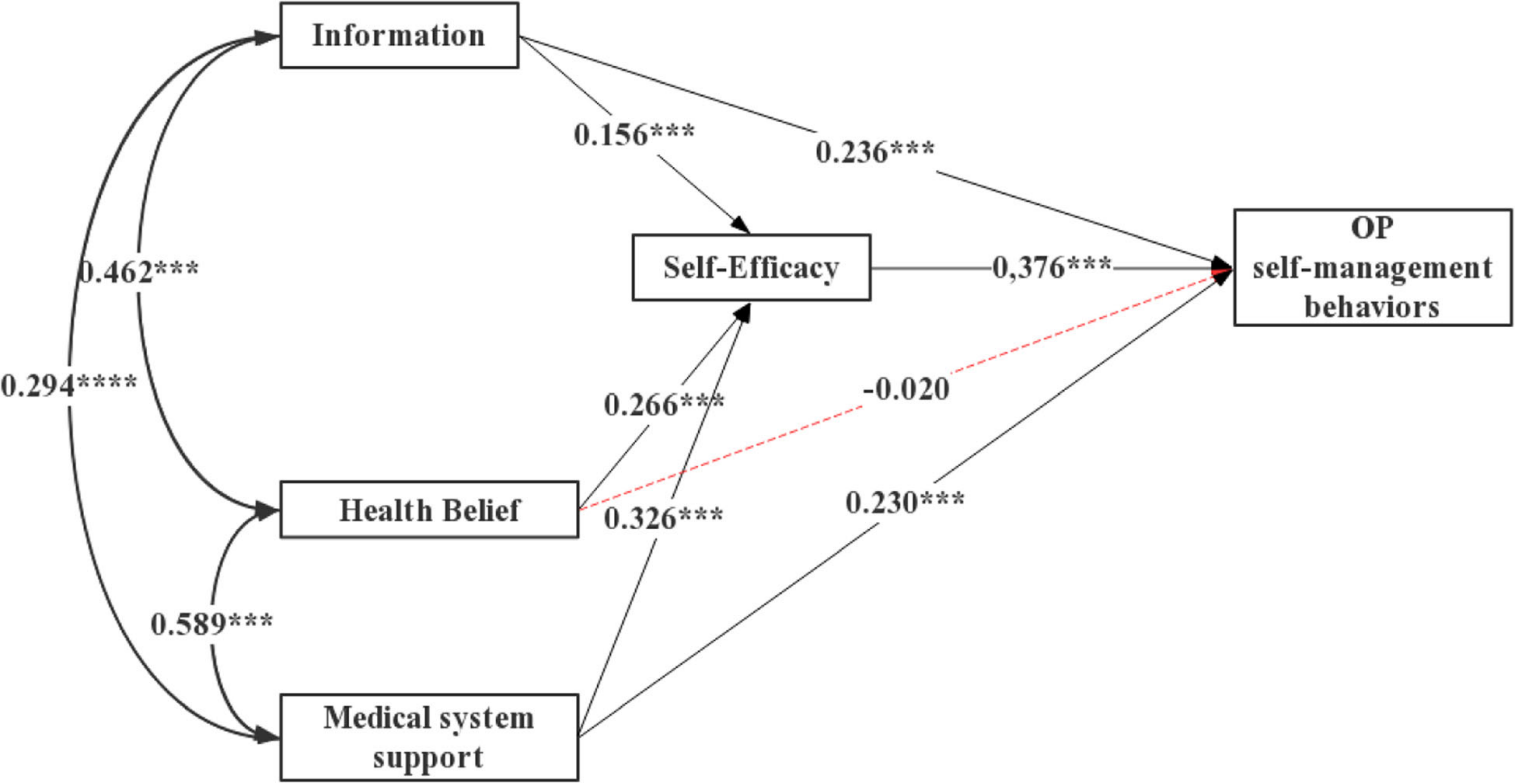
Estimation of the modified Information-Motivation-Behavioral skill model of OP self-management behaviors (*N* = 571)

Figure 1 shows the model outcomes with estimates for regression weights, correlations, and path coefficients. The results were: χ^2^ = 956.951, df = 10, *P* < 0.001, goodness of fit index (GFI) = 1.000, adjusted goodness of fit index (AGFI) = 0.998, normed fit index (NFI) = 1.000, comparative fit index (CFI) = 1.000, and root mean square error of approximation (RMSEA) = 0.000. These results indicated that the model fit the data.

The model was interpreted as follows. We estimated a model in which personal and social motivations to adhere were permitted to co-vary. Health beliefs and medical system support significantly co-varied (r = 0.589, *P* < 0.001). Information for motivation (health beliefs and medical system support) significantly co-varied (*r* = 0.462, *P* < 0.001 and r = 0.294, *P* < 0.001, respectively). Paths from information to self-efficacy (β = 0.156, *P* < 0.001) and self-management behaviors (β = 0.236, *P* < 0.001), from health beliefs to self-efficacy (β = 0.266, *P* < 0.001), from medical system support to self-efficacy (β = 0.326, *P* < 0.001) and self-management behaviors (β = 0.230, *P* < 0.001), and from self-efficacy to self-management behaviors (β = 0.376, *P* < 0.001) were significant and in the predicted direction. Paths from health beliefs to self-management behaviors (β = − 0.020, *P* = 0.586) were insignificant.

### Testing of mediation effects

The mediation test results for the relationships among information, health beliefs, medical system support, self-efficacy, and self-management behaviors are summarized in Table 3. The indirect effects of health beliefs mediated self-management behaviors through self-efficacy as presented in our final model was 0.051 (95% CI = 0.034–0.070). There were no direct effects between health beliefs and self-management behaviors. The indirect effects of information and medical system support mediated self-management behaviors through self-efficacy were 0.060 (95% CI = 0.030–0.097) and 0.142 (95% CI = 0.098–0.190), respectively.

**Table 3:** Effects of information, health beliefs, and medical system support on osteoporosis mediated through self-efficacy examined by a bias-corrected bootstrap method

Independent variable (IV)	Mediator(M)	Dependent variable (DV)	Effect of IV on M(a)	Effect of M on DV(b)	Total effect (c)	Direct effect of IV on DV (c′)	Indirect effect	
							(a × b)	95%CI
Information	Self-Efficacy	OP self-management behaviors	0.710	0.084	0.301	0.241	0.060	0.030–0.097
Health Belief			0.601		0.040	−0.010	0.051	0.034–0.070
Medical System Support			1.681		0.408	0.266	0.142	0.098–0.190

Mediation effects were tested using a bias-corrected bootstrap method in the AMOS 23.0 software. The results were non-standardized estimates

## Discussion

In this study, we examined whether the IMB model could predict OP self-management behaviors, and investigated how the model constructs influenced self-management behaviors among patients with OP. Overall, the results indicated that the IMB model-based framework for understanding self-management behaviors was well positioned to explain the sample data.

First, information, health beliefs, and medical system support were associated with self-efficacy, which in turn was correlated with self-management behaviors. This highlighted the importance of self-efficacy in health interventions for OP self-management behaviors. Self-efficacy is an individual’s ability to change their behavior, which is fundamental in reducing the incidence of chronic diseases and improving health outcomes [43]. Second, paths from information were directly associated with self-efficacy and self-management behaviors. In other diseases, inconsistencies were observed between information and preventive behavior [44]. This difference may be attributable to differences in the types of disease and levels of cognition. Infectious diseases such as AIDS are of wide concern and have a high level of cognition, whereas OP is a chronic disease that is not easily detected, and people generally lack OP-related knowledge. Information may have a significant impact on initial behavioral changes, suggesting that having better knowledge about OP may directly help Chinese patients with OP to improve their self-management behaviors. Sedlak et al. [45] found that personal knowledge of BMD obtained through DXA screening had a more powerful relationship with OP preventive behaviors than general knowledge of OP. This finding has important implications for healthcare providers. Third, paths from medical system support were directly associated with self-efficacy and self-management behaviors. Chinese patients usually rely on physician’s suggestions for disease treatment, and receive healthcare services from primary healthcare providers. This suggests that we should include OP in the standardized management of community health centers, similar to management for diabetes and high blood pressure. Fourth, health beliefs had an indirect effect on self-management behaviors through self-efficacy. A previous meta-analysis reported there were no significant predictive relationships between individual illness belief domains and adherence to self-management behaviors in 14 of 52 included papers [46]. Interactions between different dimensions of health beliefs might have influenced potential associations with adherence to self-management behaviors.

Understanding of OP has advanced in the medical community, but translation of this knowledge to the lay community has lagged behind. Patients often take a laissez-faire attitude toward OP that can affect self-management behaviors [4]. In this study, only 101 (17.7%) participants had better OP self-management behaviors. Better self-management behaviors were observed in 87 (15.2%) participants in the nutrition dimension and 122 (21.4%) participants in the exercise dimension, which was lower than the 50.4 and 38.9% for these dimensions reported for patients with diabetes in a previous study [47]. In particular, there was a large difference in the nutrition dimension in our study compared with the previous study. Therefore, nutrition labeling knowledge should be popularized in future health education efforts to help residents choose healthy foods. In general, the self-management behaviors for OP in middle-aged and older adult patients were poor. We recommend that OP should be included in the standardized management of chronic diseases in the community to help patients establish good self-management behaviors.

### Limitations

First, the cross-sectional data prevented us from drawing conclusions about true causal mediation and an ability to test the IMB model’s proposed feedback loop from health outcomes to information and motivation [34]. Second, we used convenience sampling, which limited the representativeness of our sample. We selected 20 community health service centers for an OP medical treatment management pilot study in Shanghai. The scope of the sample in this study was broad, meaning the sample reflects the actual situation in shanghai to some extent. Further extensive unbiased studies and a cohort study in a larger population are warranted to confirm the results. Third, data were gathered from a self-reported questionnaire, and consequently the reliability of responses to sensitive questions may be questionable. Fourth, because of the lack of objective skills for testing behavior in OP, the behavioral skills in this study were tested using only one dimension (self-efficacy). This research focused on exploring support from the medical system, but further research needs to consider more dimensions.

## Conclusions

Our study is the first to assess the relevance of the IMB model for OP self-management behaviors. Middle-aged and older adult patients with OP have poor self-management behavior. Medical system support can help patients develop good self-management behavior. OP-related knowledge can change patients’ cognition, help improve patients’ self-confidence (self-efficacy), and establish good self-management behaviors. Therefore, it should be considered in health management.

## Supplementary information

**Additional file 1.** Self-health management questionnaire for patients with osteoporosis.

## Data Availability

The datasets analyzed in the current study are available from the corresponding author on reasonable request.

## Abbreviations

AGFI: Adjusted Goodness of Fit Index
BMD: Bone mineral density
CFI: Comparative Fit Index
DXA: Dual-energy X-ray absorption
GFI: Goodness of Fit Index,
IMB: Information–Motivation–Behavioral Skills
NFI: Normed Fit Index
OHBS: Osteoporosis Health Belief Scale
OKT: Osteoporosis Knowledge Test
OP: Osteoporosis
OSES: Osteoporosis Self-Efficacy Scale
RMSEA: Root Mean Square Error of Approximation

## References

[1] Peck WA. Consensus development conference: diagnosis, prophylaxis, and treatment of osteoporosis. Am J Med. 1993;94(6):646–50. 10.1016/0002-9343(93)90218-e 8506892

[2] Chen P, Li Z, Hu Y (2016). Prevalence of osteoporosis in China: a meta-analysis and systematic review. BMC Public Health.

[3] Haleem S, Lutchman L, Mayahi R, Grice JE, Parker MJ (2008). Mortality following hip fracture: trends and geographical variations over the last 40 years. Injury.

[4] Si L, Winzenberg TM, Jiang Q, Chen M, Palmer AJ (2015). Projection of osteoporosis-related fractures and costs in China: 2010-2050. Osteoporos Int.

[5] Wang L, Xu X, Zhang Y, Hao H, Chen L, Su T, Zhang Y, Ma W, Xie Y, Wang T (2016). A model of health education and management for osteoporosis prevention. Exp Ther Med.

[6] Kanis JA, Cooper C, Rizzoli R, Reginster JY, Scientific Advisory Board of the European Society for C, Economic Aspects of O, the Committees of Scientific A, National Societies of the International Osteoporosis F (2019). European guidance for the diagnosis and management of osteoporosis in postmenopausal women. Osteoporos Int.

[7] Yeam CT, Chia S, Tan HCC, Kwan YH, Fong W, Seng JJB (2018). A systematic review of factors affecting medication adherence among patients with osteoporosis. Osteoporos Int.

[8] Ross S, Samuels E, Gairy K, Iqbal S, Badamgarav E, Siris E (2011). A meta-analysis of osteoporotic fracture risk with medication nonadherence. Value Health.

[9] Huybrechts KF, Ishak KJ, Caro JJ (2006). Assessment of compliance with osteoporosis treatment and its consequences in a managed care population. Bone.

[10] Rabenda V, Mertens R, Fabri V, Vanoverloop J, Sumkay F, Vannecke C, Deswaef A, Verpooten GA, Reginster JY (2008). Adherence to bisphosphonates therapy and hip fracture risk in osteoporotic women. Osteoporos Int.

[11] Peterlik M, Kallay E, Cross HS (2013). Calcium nutrition and extracellular calcium sensing: relevance for the pathogenesis of osteoporosis, cancer and cardiovascular diseases. Nutrients.

[12] Ryan P, Sawin KJ (2009). The individual and family self-management theory: background and perspectives on context, process, and outcomes. Nurs Outlook.

[13] Barlow J, Wright C, Sheasby J, Turner A, Hainsworth J (2002). Self-management approaches for people with chronic conditions: a review. Patient Educ Couns.

[14] Newman S, Steed L, Mulligan K (2004). Self-management interventions for chronic illness. Lancet.

[15] Ryan P (2009). Integrated theory of health behavior change: background and intervention development. Clin Nurse Spec.

[16] Lee G, Yang SJ, Chee YK (2016). Assessment of healthy behaviors for metabolic syndrome among Korean adults: a modified information-motivation-behavioral skills with psychological distress. BMC Public Health.

[17] Fallahi A, Derakhshan S, Pashaee T, Teymoori P (2015). Factors affecting self-care in women with osteoporosis: a qualitative study with the content analysis approach. J Sch Public Health Inst Public Health Res.

[18] Evenson AL, Sanders GF (2016). Educational intervention impact on osteoporosis knowledge, health beliefs, self-efficacy, dietary calcium, and vitamin D intakes in young adults. Orthop Nurs.

[19] Malekshahi F, Hidarnia A, Niknami S, Aminshokravi F (2015). The determination of predictive construct of physical behavior change on osteoporosis prevention women aged 30-50: a trans-theoretical method study. Global J Health Sci.

[20] Park KS, Yoo JI, Kim HY, Jang S, Park Y, Ha YC (2017). Education and exercise program improves osteoporosis knowledge and changes calcium and vitamin D dietary intake in community dwelling elderly. BMC Public Health.

[21] Mayberry LS, Osborn CY (2014). Empirical validation of the information-motivation-behavioral skills model of diabetes medication adherence: a framework for intervention. Diabetes Care.

[22] Prochaska JO, DiClemente CC, Norcross JC (1992). In search of how people change. Applications to addictive behaviors. Am Psychol.

[23] Carpenter CJ (2010). A meta-analysis of the effectiveness of health belief model variables in predicting behavior. Health Commun.

[24] Janz NK, Becker MH (1984). The health belief model: a decade later. Health Educ Q.

[25] Osborn CY, Fisher JD (2008). Diabetes education: integrating theory, cultural considerations, and individually tailored content. Clin Diabetes.

[26] Jeihooni AK, Hidarnia A, Kaveh MH, Hajizadeh E, Askari A (2015). Effects of an osteoporosis prevention program based on health belief model among females. Nurs Midwifery Stud.

[27] Alamri FA, Saeedi MY, Mohamed A, Barzanii A, Aldayel M, Ibrahim AK (2015). Knowledge, attitude, and practice of osteoporosis among Saudis: a community-based study. J Egypt Public Health Assoc.

[28] Ievers-Landis CE, Burant C, Drotar D, Morgan L, Trapl ES, Kwoh CK (2003). Social support, knowledge, and self-efficacy as correlates of osteoporosis preventive behaviors among preadolescent females. J Pediatr Psychol.

[29] Zomahoun HTV, Guenette L, Gregoire JP, Lauzier S, Lawani AM, Ferdynus C, Huiart L, Moisan J (2017). Effectiveness of motivational interviewing interventions on medication adherence in adults with chronic diseases: a systematic review and meta-analysis. Int J Epidemiol.

[30] Osborn CY, Egede LE (2010). Validation of an information-motivation-behavioral skills model of diabetes self-care (IMB-DSC). Patient Educ Couns.

[31] Fisher JD, Fisher WA (1992). Changing AIDS-risk behavior. Psychol Bull.

[32] Jeihooni AK, Hidarnia A, Kaveh MH, Hajizadeh E, Askari A (2016). Application of the health belief model and social cognitive theory for osteoporosis preventive nutritional behaviors in a sample of Iranian women. Iran J Nurs Midwifery Res.

[33] Chen Y, Zou H, Zhang Y, Fang W, Fan X (2017). Family caregiver contribution to self-care of heart failure: an application of the information-motivation-behavioral skills model. J Cardiovasc Nurs.

[34] Fisher JD, Fisher WA, Amico KR, Harman JJ (2006). An information-motivation-behavioral skills model of adherence to antiretroviral therapy. Health Psychol.

[35] Khani Jeihooni A, Hidarnia A, Kaveh MH, Hajizadeh E (2015). The effect of a prevention program based on health belief model on osteoporosis. J Res Health Sci.

[36] Mueller RO (1997). Structural equation modeling: Back to basics. Struct Equ Model Multidiscip J.

[37] Thompson B (2000). Ten commandments of structural equation modeling. Reading and understanding MORE multivariate statistics.

[38] Kim KK, Horan ML, Gendler P, Patel MK (1991). Development and evaluation of the osteoporosis health belief scale. Res Nurs Health.

[39] Maningat P, Gordon BR, Breslow JL (2013). How do we improve patient compliance and adherence to long-term statin therapy?. Curr Atheroscler Rep.

[40] Horan ML, Kim KK, Gendler P, Froman RD, Patel MD (1998). Development and evaluation of the osteoporosis self-efficacy scale. Res Nurs Health.

[41] Soleymanian A, Niknami S, Hajizadeh E, Shojaeizadeh D, Montazeri A (2014). Development and validation of a health belief model based instrument for measuring factors influencing exercise behaviors to prevent osteoporosis in pre-menopausal women (HOPE). BMC Musculoskelet Disord.

[42] Shen QM, Shen T, Wang ZZ, Shi Y, Lhakpa T, Yang YH, Wang HP, Cai Y, Shang ML (2018). Evaluation of validity and reliability of self-management behavior scale for community patients with osteoporosis. Chinese J Gen Pract.

[43] Fleig L, Pomp S, Schwarzer R, Lippke S (2013). Promoting exercise maintenance: how interventions with booster sessions improve long-term rehabilitation outcomes. Rehabil Psychol.

[44] Zhang H, Liao M, Nie X, Pan R, Wang C, Ruan S, Zhang C, Tao X, Kang D, Jiang B (2011). Predictors of consistent condom use based on the information-motivation-behavioral skills (IMB) model among female sex workers in Jinan, China. BMC Public Health.

[45] Sedlak CA, Doheny MO, Estok PJ, Zeller RA, Winchell J (2007). DXA, health beliefs, and osteoporosis prevention behaviors. J Aging Health.

[46] Aujla N, Walker M, Sprigg N, Abrams K, Massey A, Vedhara K (2016). Can illness beliefs, from the common-sense model, prospectively predict adherence to self-management behaviours? A systematic review and meta-analysis. Psychol Health.

[47] Zhang X, Shiyan W U, Wang F, et al. Association between social support and self-management behaviors among patients with diabetes in community[J]. Journal of Peking University (Health Sciences). 2017;49(3):455–61. 28628147

