# Supplementary material for: Applying the information–motivation–behavioral model to explore the influencing factors of self-management behavior among osteoporosis patients
Source: BMC Public Health. 2020 Feb 6;20:198. doi: 10.1186/s12889-020-8292-x (PMC7006415; doi:10.1186/s12889-020-8292-x)
Supplement: Supplementary file 1 — Additional file 1. Self-health management questionnaire for patients with osteoporosis. [file 12889_2020_8292_MOESM1_ESM.docx]

Community  number:

**Self-health management questionnaire for patients with osteoporosis**

Dear friend, hello!

This is a questionnaire about bone health and I hope to get your support. We will keep your personal information confidential, I hope you do not have any concerns, fill in the truth, ensure that the information is true and effective, please read the questions carefully, fill in the relevant basic information, and use the pen to answer the answer to the corresponding survey questions. All the unspecified questions are single-choice questions, just choose one answer, thank you for your cooperation!

**A. demographic characteristics**

A1. Birthday  (Y) (M)

A2. Gender (1) male (2) female

A3. Height  (CM)

A4. Weight    (KG)

A5. Your education level:

(1) Elementary school and below (2) Junior high school

(3) High school (secondary / vocational school)

(4) Junior college (5) Bachelor degree or above

A6. Your marital status

(1) Unmarried (2) Married

(3) Divorced (4) Widowed

A7.Your monthly income (RMB: Yuan)

(1)3000 and below (2)3001-6000   (3)6001-10000  (4)10000 or more

A8. Is there a family history of osteoporosis in your home?

(1)  Yes (2 ) No

A9. How long do you have osteoporosis(year)

1. None (2) <5 years (3) 5-9 years

(4)≥10 years

**B.** **Below are some information related to osteoporosis. Please judge whether it is correct or you don't know. Please type “√”or draw “ ○ ”on the corresponding number of each question.**

| **Patient's osteoporosis knowledge problem** | **Correct** | **wrong** | **do not know** |
| --- | --- | --- | --- |
| 1. Usually eating less dairy products ( less milk ) is more likely to suffer from osteoporosis. | 1 | 2 | 3 |
| 2. Menopausal women are not prone to osteoporosis. | 1 | 2 | 3 |
| 3. Parents/(external) grandparents with osteoporosis are more likely to have osteoporosis. | 1 | 2 | 3 |
| 4. Caucasian or Asian women are not prone to osteoporosis. | 1 | 2 | 3 |
| 5.The elderly men are less likely to get osteoporosis than women. | 1 | 2 | 3 |
| 6. People who have had an oophorectomy are less likely to get osteoporosis. | 1 | 2 | 3 |
| 7. Hormonal agents taking a long time (Such as cortisone) are more difficult to get osteoporosis . | 1 | 2 | 3 |
| 8. People who are overweight are more likely to get osteoporosis. | 1 | 2 | 3 |
| 9. People eating disorders are more prone to bone osteoporosis . | 1 | 2 | 3 |
| 10. People who drink at least two glasses of wine a day are not prone to osteoporosis. | 1 | 2 | 3 |
| 11. People who smoke every day are less likely to get osteoporosis. | 1 | 2 | 3 |
| 12. Exercise once a week to strengthen the bones. | 1 | 2 | 3 |
| 13. Exercise can make bones strong regardless of exercise intensity. | 1 | 2 | 3 |
| 14. Quick walking can reduce the chance of suffering from a fracture . | 1 | 2 | 3 |
| 15. Weight-bearing exercise can reduce the risk of osteoporosis. | 1 | 2 | 3 |
| 16. Jogging can reduce the chance of suffering from osteoporosis. | 1 | 2 | 3 |
| 17. Aerobic exercise such as square dance can reduce the risk of osteoporosis. | 1 | 2 | 3 |
| 18. Soy is a good source of calcium. | 1 | 2 | 3 |
| 19. Shrimp is a good source of calcium. | 1 | 2 | 3 |
| 20. Broccoli is a good source of calcium. | 1 | 2 | 3 |
| 21. Yogurt is a good source of calcium. | 1 | 2 | 3 |
| 22. bean curd is a good source of calcium. | 1 | 2 | 3 |
| 23. The adult daily recommended intake of calcium is 4 00- 600 mg / day. | 1 | 2 | 3 |
| 24. In order to meet the recommended daily intake of calcium for adults, drink at least 500ml(2 cups) of milk. | 1 | 2 | 3 |
| 25. If you can't get enough calcium from your diet, you should take calcium tablets . | 1 | 2 | 3 |
| 26. Vitamin A is an essential vitamin for calcium absorption. | 1 | 2 | 3 |
| 27. Regular exposure to sunlight promotes the synthesis of vitamin D, which helps calcium absorption. | 1 | 2 | 3 |
| 28. Cod liver oil provides vitamins that help calcium absorption better. | 1 | 2 | 3 |
| 29. Adults over 50 years old need to take vitamin D 800-1000IU/ 20 - 25mg every day. | 1 | 2 | 3 |
| 30. Adulthood is the best time to shape strong bones . | 1 | 2 | 3 |
| 3 1. Osteoporosis can be double an amount of radiation absorbed bone densitometer diagnostic test . | 1 | 2 | 3 |
| 32. The drug can treat osteoporosis. | 1 | 2 | 3 |

**C. belief**

|  | **Strongly disagree** | **Disagree** | **Neutral** | **Agree** | **Strongly Agree** |
| --- | --- | --- | --- | --- | --- |
| **Susceptibility** |  |  |  |  |  |
| 1. Your chances of getting osteoporosis are high. | 1 | 2 | 3 | 4 | 5 |
| 2. Because of your body build, you are more likely to develop osteoporosis. | 1 | 2 | 3 | 4 | 5 |
| 3. It is extremely likely that you will get osteoporosis. | 1 | 2 | 3 | 4 | 5 |
| 4. There is a good chance that you will get osteoporosis. | 1 | 2 | 3 | 4 | 5 |
| 5. You are more likely than the average person to get osteoporosis. | 1 | 2 | 3 | 4 | 5 |
| 6. Your family history makes it more likely that you will get osteoporosis. | 1 | 2 | 3 | 4 | 5 |
| **Severity** |  |  |  |  |  |
| 7. The thought of having osteoporosis scares you. | 1 | 2 | 3 | 4 | 5 |
| 8. If you had osteoporosis you would be crippled. | 1 | 2 | 3 | 4 | 5 |
| 9. Your feelings about yourself would change if you got osteoporosis. | 1 | 2 | 3 | 4 | 5 |
| 10. It would be very costly if you got osteoporosis. | 1 | 2 | 3 | 4 | 5 |
| 11. When you think about osteoporosis you get depressed. | 1 | 2 | 3 | 4 | 5 |
| 12. It would be very serious if you got osteoporosis. | 1 | 2 | 3 | 4 | 5 |
| **Benefits of exercise** |  |  |  |  |  |
| 13. Regular exercise prevents problems that would happen from osteoporosis. | 1 | 2 | 3 | 4 | 5 |
| 14. You feel better when you exercise to prevent osteoporosis. | 1 | 2 | 3 | 4 | 5 |
| 15. Regular exercise helps to build strong bones. | 1 | 2 | 3 | 4 | 5 |
| 16. Exercising to prevent osteoporosis also improves the way your body looks. | 1 | 2 | 3 | 4 | 5 |
| 17. Regular exercise cuts down the chances of broken bones. | 1 | 2 | 3 | 4 | 5 |
| 18. You feel good about yourself when you exercise to prevent osteoporosis. | 1 | 2 | 3 | 4 | 5 |
| **Benefits of calcium supplementation** |  |  |  |  |  |
| 19. Taking in enough calcium prevents problems from osteoporosis. | 1 | 2 | 3 | 4 | 5 |
| 20. You have lots to gain from taking in enough calcium to prevent osteoporosis. | 1 | 2 | 3 | 4 | 5 |
| 21. Taking in enough calcium prevents painful osteoporosis. | 1 | 2 | 3 | 4 | 5 |
| 22. You would not worry as much about osteoporosis if you took in enough calcium. | 1 | 2 | 3 | 4 | 5 |
| 23. Taking in enough calcium cuts down on your chances of broken bones. | 1 | 2 | 3 | 4 | 5 |
| 24. You feel good about yourself when you take in enough calcium to prevent osteoporosis. | 1 | 2 | 3 | 4 | 5 |
| **Difficulties and obstacles to exercise** |  |  |  |  |  |
| 25. You feel like you are not strong enough to exercise regularly. | 1 | 2 | 3 | 4 | 5 |
| 26. You have no place where you can exercise | 1 | 2 | 3 | 4 | 5 |
| 27. Your spouse or family discourages you from exercising. | 1 | 2 | 3 | 4 | 5 |
| 28. Exercising regularly would mean starting a new habit which is hard for you to do. | 1 | 2 | 3 | 4 | 5 |
| 29. Exercising regularly makes you uncomfortable. | 1 | 2 | 3 | 4 | 5 |
| 30. Exercising regularly upsets your every day routine. | 1 | 2 | 3 | 4 | 5 |
| **Difficulties in supplementing calcium** |  |  |  |  |  |
| 31. Calcium rich foods do not agree with you. | 1 | 2 | 3 | 4 | 5 |
| 32. You do not like calcium rich foods. | 1 | 2 | 3 | 4 | 5 |
| 33. You don't like foods rich in calcium. | 1 | 2 | 3 | 4 | 5 |
| 34. Eating calcium rich foods means changing your diet which is hard to do. | 1 | 2 | 3 | 4 | 5 |
| 35. In order to eat more calcium-rich foods, you have to give up other foods you like. | 1 | 2 | 3 | 4 | 5 |
| 36. Calcium rich foods have too much cholesterol. | 1 | 2 | 3 | 4 | 5 |
| **Osteoporosis Prevention of contributing factor** |  |  |  |  |  |
| 37. You eat a well-balanced diet. | 1 | 2 | 3 | 4 | 5 |
| 38. You look for new information related to health. | 1 | 2 | 3 | 4 | 5 |
| 39. Keeping healthy is very important for you. | 1 | 2 | 3 | 4 | 5 |
| 40. You try to discover health problems early | 1 | 2 | 3 | 4 | 5 |
| 41. You have a regular health check-up even when you are not sick. | 1 | 2 | 3 | 4 | 5 |
| 42. You follow recommendations to keep you healthy. | 1 | 2 | 3 | 4 | 5 |

**D. Osteoporosis self-efficacy If with a Zhou as a unit, you are advised to do the following few things that you can no do it? Please circle a number in each line according to your actual situation, indicating how much you can do it.**

| **I am confident :** | **Not at all → May be May be not → Can do it all** | | | | | | | | | | |
| --- | --- | --- | --- | --- | --- | --- | --- | --- | --- | --- | --- |
| 1. begin a new or different exercise program | 0 | 1 | 2 | 3 | 4 | 5 | 6 | 7 | 8 | 9 | 10 |
| 2. Change your exercise habits. | 0 | 1 | 2 | 3 | 4 | 5 | 6 | 7 | 8 | 9 | 10 |
| 3. put forth the effort required to exercise. | 0 | 1 | 2 | 3 | 4 | 5 | 6 | 7 | 8 | 9 | 10 |
| 4. do exercises even if they are difficult. | 0 | 1 | 2 | 3 | 4 | 5 | 6 | 7 | 8 | 9 | 10 |
| 5. Maintain a regular daily exercise program. | 0 | 1 | 2 | 3 | 4 | 5 | 6 | 7 | 8 | 9 | 10 |
| 6. exercise for the appropriate length of time. | 0 | 1 | 2 | 3 | 4 | 5 | 6 | 7 | 8 | 9 | 10 |
| 7. do exercises even if they are tiring | 0 | 1 | 2 | 3 | 4 | 5 | 6 | 7 | 8 | 9 | 10 |
| 8. stick to your exercise program. | 0 | 1 | 2 | 3 | 4 | 5 | 6 | 7 | 8 | 9 | 10 |
| 9. exercise at least three times a week . | 0 | 1 | 2 | 3 | 4 | 5 | 6 | 7 | 8 | 9 | 10 |
| 10. do the type of exercises that you are supposed to do. | 0 | 1 | 2 | 3 | 4 | 5 | 6 | 7 | 8 | 9 | 10 |
| 11. begin to eat more calcium rich foods. | 0 | 1 | 2 | 3 | 4 | 5 | 6 | 7 | 8 | 9 | 10 |
| 12. Increase calcium intake . | 0 | 1 | 2 | 3 | 4 | 5 | 6 | 7 | 8 | 9 | 10 |
| 13. consume adequate amounts of calcium rich foods. | 0 | 1 | 2 | 3 | 4 | 5 | 6 | 7 | 8 | 9 | 10 |
| 14. eat calcium rich foods on a regular basis. | 0 | 1 | 2 | 3 | 4 | 5 | 6 | 7 | 8 | 9 | 10 |
| 15. change your diet to include more calcium rich foods | 0 | 1 | 2 | 3 | 4 | 5 | 6 | 7 | 8 | 9 | 10 |
| 16. eat calcium rich foods as often as you are supposed to do | 0 | 1 | 2 | 3 | 4 | 5 | 6 | 7 | 8 | 9 | 10 |
| 17. select appropriate foods to increase your calcium intake. | 0 | 1 | 2 | 3 | 4 | 5 | 6 | 7 | 8 | 9 | 10 |
| 18. stick to a diet which gives an adequate amount of calcium. | 0 | 1 | 2 | 3 | 4 | 5 | 6 | 7 | 8 | 9 | 10 |
| 19. obtain foods that give an adequate amount of calcium. | 0 | 1 | 2 | 3 | 4 | 5 | 6 | 7 | 8 | 9 | 10 |
| 20. remember to eat calcium rich foods. | 0 | 1 | 2 | 3 | 4 | 5 | 6 | 7 | 8 | 9 | 10 |
| 21. take calcium supplements if you don’t get enough calcium from you diet. | 0 | 1 | 2 | 3 | 4 | 5 | 6 | 7 | 8 | 9 | 10 |

**E** **Medical system support for patients**

|  | **Strongly disagree** | **Disagree** | **Neutral** | **Agree** | **Strongly Agree** |
| --- | --- | --- | --- | --- | --- |
| 1. As you know, hospitals and community clinics for osteoporosis have established health records for you. | 1 | 2 | 3 | 4 | 5 |
| 2. According to your knowledge, experts from higher-level hospitals enter the community to conduct osteoporosis lectures no less than twice a year. | 1 | 2 | 3 | 4 | 5 |
| 3. If you need it, your doctor can provide you with referral information. | 1 | 2 | 3 | 4 | 5 |
| 4.The doctor gave me confidence. | 1 | 2 | 3 | 4 | 5 |
| 5.The doctor is willing to help me. | 1 | 2 | 3 | 4 | 5 |
| 6.I think the doctor is trustworthy. | 1 | 2 | 3 | 4 | 5 |
| 7.The doctor answered my questions in great detail. | 1 | 2 | 3 | 4 | 5 |
| 8. I can get information about osteoporosis from doctor. | 1 | 2 | 3 | 4 | 5 |
| 9. The doctor answered my question in time.. | 1 | 2 | 3 | 4 | 5 |
| 10. The doctor listened to me carefully. | 1 | 2 | 3 | 4 | 5 |
| 11. The doctor is very patient with me. | 1 | 2 | 3 | 4 | 5 |

**I .Osteoporosis self-management behavior**

**Please tick the frequency of the following behaviors you have completed in the past 30 days.**

|  | **Never** | **Very few** | **Occasionally** | **most** | **often** |
| --- | --- | --- | --- | --- | --- |
| 1.Pay attention to reading the calcium content on the food ingredient label. | 1 | 2 | 3 | 4 | 5 |
| 2.Pay attention to reading the vitamin D content on the food ingredient label. | 1 | 2 | 3 | 4 | 5 |
| 3.Choose high calcium foods to maintain a healthy diet plan (such as shrimp, soy). | 1 | 2 | 3 | 4 | 5 |
| 4.Choose a high vitamin D content to maintain a diet plan (such as cod liver oil, spinach). | 1 | 2 | 3 | 4 | 5 |
| 5. Maintain aerobic exercise three times a week or more (such as brisk walking, aerobics), each time lasting 30 minutes or more | 1 | 2 | 3 | 4 | 5 |
| 8.Follow the set exercise plan. | 1 | 2 | 3 | 4 | 5 |
| 9.Proactively go to the osteoporosis clinic according to the doctor's time or self-test bone density. | 1 | 2 | 3 | 4 | 5 |
| 10.Can easily and accurately express your symptoms to doctors. | 1 | 2 | 3 | 4 | 5 |
| 11.Be patient and listen carefully to doctor's statement. | 1 | 2 | 3 | 4 | 5 |
| 12.Ask your doctor when you have doubts. | 1 | 2 | 3 | 4 | 5 |
| 13.Follow the doctor's advice to take calcium or drugs. | 1 | 2 | 3 | 4 | 5 |
